# An Experimental Model of Bronchopulmonary Dysplasia Features Long-Term Retinal and Pulmonary Defects but Not Sustained Lung Inflammation

**DOI:** 10.3389/fped.2021.689699

**Published:** 2021-08-30

**Authors:** Lakshanie C. Wickramasinghe, Peter van Wijngaarden, Chad Johnson, Evelyn Tsantikos, Margaret L. Hibbs

**Affiliations:** ^1^Leukocyte Signalling Laboratory, Department of Immunology and Pathology, Central Clinical School, Monash University, Melbourne, VIC, Australia; ^2^Department of Surgery - Ophthalmology, University of Melbourne, Melbourne, VIC, Australia; ^3^Centre for Eye Research Australia, Royal Victorian Eye and Ear Hospital, East Melbourne, VIC, Australia; ^4^Monash Micro Imaging, Alfred Research Alliance, Monash University, Melbourne, VIC, Australia

**Keywords:** bronchopulmonary dysplasia, lung development, inflammation, animal model, supplemental oxygen, chronic obstructive pulmonary disease, retinopathy of prematurity, choroidal thinning

## Abstract

Bronchopulmonary dysplasia (BPD) is a severe lung disease that affects preterm infants receiving oxygen therapy. No standardized, clinically-relevant BPD model exists, hampering efforts to understand and treat this disease. This study aimed to evaluate and confirm a candidate model of acute and chronic BPD, based on exposure of neonatal mice to a high oxygen environment during key lung developmental stages affected in preterm infants with BPD. Neonatal C57BL/6 mouse pups were exposed to 75% oxygen from postnatal day (PN)-1 for 5, 8, or 14 days, and their lungs were examined at PN14 and PN40. While all mice showed some degree of lung damage, mice exposed to hyperoxia for 8 or 14 days exhibited the greatest septal wall thickening and airspace enlargement. Furthermore, when assessed at PN40, mice exposed for 8 or 14 days to supplemental oxygen exhibited augmented septal wall thickness and emphysema, with the severity increased with the longer exposure, which translated into a decline in respiratory function at PN80 in the 14-day model. In addition to this, mice exposed to hyperoxia for 8 days showed significant expansion of alveolar epithelial type II cells as well as the greatest fibrosis when assessed at PN40 suggesting a healing response, which was not seen in mice exposed to high oxygen for a longer period. While evidence of lung inflammation was apparent at PN14, chronic inflammation was absent from all three models. Finally, exposure to high oxygen for 14 days also induced concurrent outer retinal degeneration. This study shows that early postnatal exposure to high oxygen generates hallmark acute and chronic pathologies in mice that highlights its use as a translational model of BPD.

## Introduction

Bronchopulmonary dysplasia (BPD) is the most common respiratory disorder affecting premature infants provided long-term oxygen therapy and respiratory support (1). Advancements in neonatal care have improved the survival of severely premature infants born earlier in gestation; however, the incidence of BPD has remained unchanged over the last decade (2). Surfactant therapy and corticosteroid treatments, in concert with less invasive respiratory support, have helped transform the lung phenotype observed in “old BPD” from extensive parenchymal fibrosis, interstitial edema and severe inflammation to a milder lung pathology observed in infants born today (3). The lung phenotype observed in infants that come into the neonatal intensive care unit (NICU) nowadays is characterized by impaired alveolar and vascular development and mild inflammation and fibrosis. Of greatest concern, is the capacity of this disease to progress into severe lung complications by adulthood, including reduced exercise capacity (4), childhood and adolescent asthma (5), and in serious cases, the development of chronic obstructive pulmonary disease (COPD) (6, 7).

Numerous high oxygen schemes have been used to model BPD in rodents. These models have contributed to our understanding of mechanisms responsible for the development of this condition, such as the role of oxidative stress and inflammation (8), and they have replicated some of the histological aberrations that occur within the human neonatal lung following the routine use of supplemental oxygen in the clinical setting (9). However, despite these findings, no single experimental model is used consistently. Previous models have used varying concentrations and durations of oxygen exposure. In most cases, there is no rationale for why a particular model was selected over others and whether concurrent changes in other tissues occur and disease persists into adulthood. Therefore, having a standardized experimental protocol of oxygen-induced BPD would limit the variability in data output between studies and most importantly, minimize discrepancies when trialing potential therapeutic candidates (10–12).

In previous studies, supraphysiological levels of oxygen (85–100%) have been administered over a long period of time (13, 14). Such high concentrations have been shown to increase the susceptibility to respiratory infections when recovering in room air (13). In addition, some mouse strains, such as FVB/N, are more susceptible to oxygen toxicity than others and therefore, sublethal oxygen concentrations can result in exaggerated lung pathology or in severe cases, death of mice in the litter (14). Even with the use of lower oxygen concentrations (40–65%), the length of oxygen exposure can also generate varying degrees of lung damage (15, 16). For instance, the use of oxygen concentrations as low as 40% administered over 7 days in the postnatal period has been shown to trigger a reduction in the total number of alveoli and an increase in respiratory resistance (15). Similarly, an oxygen concentration of 65% delivered over 1 month has also been shown to impair alveolar structure (16). In a 2015 review, it was noted that in a 30-month period alone, there were 41 publications that used different strategies to model BPD, highlighting the need to establish a model that would provide consistency and standardization across neonatal respiratory research studies (12). More recently, a comprehensive multi-model study tested oxygen concentrations of 40, 60, and 80% over 14 days, as well as a 24 h oscillating exposure that ranged from 85 to 40% (15). This study found that the use of 85% oxygen for 7 or 14 days from birth caused the most lung damage, inducing septal wall thickness and alveolar enlargement (15), which are two key features of “new” BPD. However, lung inflammation and fibrosis were not examined in this study, with the extent of both being an important distinguishing feature between the “old” form of BPD and “new” BPD. In addition, the long-term consequence of early life oxygen-induced injury in the adult period was not investigated, which is an essential component of any BPD model to aid in the understanding of the deleterious effect of oxygen therapy on long-term sequelae in the lung. Another important consideration is the capacity of a BPD model to induce concurrent retinal damage, given that in a clinical setting, retinopathy of prematurity (ROP) frequently presents as a co-morbidity of BPD (17). Therefore, an oxygen protocol modeling BPD that could also incite the development of other neonatal diseases that affect a preterm infant could enhance the clinical relevance of the model, as well as its pre-clinical utility.

The appearance of the alveolar deformities associated with BPD is largely connected with the interruption to normal lung development (8). This is almost certainly due to the fact that the majority of premature babies are born during the final two stages of lung organogenesis—the saccular and alveolar stages—the time at which they receive oxygen therapy. The saccular stage prepares the lung for life outside of the womb, with the formation of primitive alveoli called saccules and production of surfactants in cuboidal alveolar cells (18–20). Simultaneously, microvascular maturation in the parenchyma leads to the thinning of the double capillary layer into a single layer, necessary to meet the respiratory requirements of the growing fetus (18, 21, 22). In the final alveolar stage of organogenesis, the saccules undergo rapid subdivision (secondary septation) into smaller gas-exchange units called alveoli, expanding the gas-exchange surface area (23, 24). In mice, the saccular stage of lung development occurs in the first 5 days after birth, which is ordinarily complete in full-term infants (25). Thus, the fact that the last two lung developmental stages occur post-birth in mice renders mice an excellent experimental tool for BPD studies.

The purpose of this study was to validate whether acute high oxygen exposure received during key periods of lung development would lead to long-term pulmonary changes. Given that the aforementioned multi-model study only assessed lung damage at day 14, in this study, we have focused on evaluating different parameters of lung pathology that may arise in adulthood, and unlike other multi-model studies, the assessment of outer retinal changes as a common co-morbidity of BPD were also prioritized in this study. Herein, we demonstrate that prolonged oxygen exposure in early neonatal life leads to sustained alveolar deterioration in adulthood, as well as defects in retinal tissue, and therefore is a suitable model for early and later life studies of BPD and research into the long-term impact of oxygen toxicity to the eye.

## Materials and Methods

### Experimental Models

C57BL/6 mice were used for this study and were purchased from Alfred Animal Services at the Alfred Research Alliance. Neonatal mice together with their dam, in litter sizes of 6–7 pups (to control for maternal nutrition), were exposed to 75% oxygen within 12 h of birth (defined as PN1) up to PN5, PN8, or PN14, and were cycled with 21% oxygen (room air) for 3 h per day to prevent oxygen toxicity to the dam (17). Following exposure, the mice were returned to room air and lungs were analyzed on PN14, PN40, and PN80. Neonatal pups and dams housed under room air conditions in the same experimental room, served as age-matched controls. All experiments were performed in accordance with National Health and Medical Research Council of Australia (NH&MRC) guidelines for animal experimentation, with ethics approval granted from the Alfred Research Alliance Animal Ethics Committee (experimental approval number: E/1746/2017/M).

### Lung Histology and Immunohistochemistry

On PN14, PN40, and PN80, postmortem lungs were inflation-fixed with 10% neutral buffered formalin at 25 cm water pressure, embedded in paraffin and sectioned by microtome at 5 μm thickness prior to staining. Lung sections were stained with Hematoxylin and Eosin (H&E) or Picrosirius Red (PR). H&E stained sections were imaged using an Olympus BX-51 bright field microscope equipped with a DP-70 color camera and 10x and 40x objectives (Olympus Corporation, Tokyo, Japan). Quantitation of alveolar airspace diameter and septal wall thickness was determined using the mean linear intercept method and alveolar septal wall thickness technique, as previously described (17). Investigators were blinded to experimental groups. PR-stained sections were scanned using the Aperio ScanScope CS (Leica Biosystems, Wetzlar, Germany) whole slide scanner at 8x and 4x magnification. Images were uploaded into ImageJ Analysis software (1.37 (NIH, Bethesda, MD; http://imagej.nih.gov/ij) and analyzed using a published script (17) with minor modifications as follows. The script used a pre-defined color threshold (set by eye) to isolate the PR signal and created a binarized image, which was measured for area. A similar method was then used to threshold the background of the tissue, in order for the total tissue area within the image to be calculated. These results were used to calculate the % PR-stained area within the tissue.

For immunohistochemistry, deparaffinized sections underwent antigen-retrieval with DAKO Target Retrieval Solution (DAKO Corp, CA, USA). Sections were blocked for 30 min with 5% BSA and incubated for 3 h at room temperature with 1:500 dilution of anti-mouse CD45 (rabbit IgG, catalog; ab10558, Abcam, Cam, UK) to stain all immune cells. Control sections had primary antibody substituted with PBS. Staining was revealed by incubation with 1:500 HRP-conjugated secondary antibody (goat anti-rabbit IgG H+L, catalog; ab205718, Abcam, Cam, UK) for 1 h and color development with diaminobenzidine chromogen solution (Agilent, CA, USA). Slides were counterstained with hematoxylin. Cells expressing CD45 were labeled brown, while negative cells were stained blue. Four randomly selected microscopic fields (40x) of lung tissue per mouse were used to calculate the percentage of CD45 positive cells/total cells as described (17). On separate lung sections, type II alveolar epithelial cells (AEC-II) were detected by staining for Pro-SPC using a previously described method (26). AEC-II's were labeled with anti-mouse Pro-SPC (rabbit IgG, 1:500, catalog ab90716: Abcam, Cam, UK), followed by red fluorescent secondary antibody (AF568 donkey anti-rabbit IgG (H+L), 1:1,000, catalog ab175693; Abcam, Cam, UK). Control sections had secondary antibody only incubated to test for non-specific binding. Stained cells were imaged using a Nikon A1r inverted confocal microscope (Nikon Corporation, Tokyo, Japan). Two large scanned images at 20x magnification were taken at two randomly selected lung sites and analyzed using a bespoke ImageJ script (Supplementary Table 1). Briefly, quantitation of AEC-II numbers was conducted with the aid of a DAPI nuclear stain as well as the overall background fluorescence to segment a field of individual cells and create regions of interest (ROIs) for each fluorescent marker. These ROIs were then used to determine if each cell was positive for a specific color based on a user defined pixel intensity threshold for each channel set up at the start of the script.

### Lung Function Assessment

At PN80, mice were anesthetized by intraperitoneal injection of 125 mg/kg of Ketamine and 10 mg/kg Xylazine (Centravet, FR). Following confirmation of deep anesthesia, mice underwent tracheostomy and a 19G cannula was inserted before being connected to an animal ventilator (Flexivent, SCIREQ, CA). Mice were mechanically ventilated (150 breaths/min, tidal volume 10 ml/kg) and a positive end-expiratory pressure (PEEP) was set at 3 cm H_2_O. The flexiware software (v8.0.4) was used to perform respiratory system mechanics, as previously detailed (27). A negative pressure-driven forced expiratory (NPFE) maneuver was performed to generate the flow-volume loop used to calculate the forced expired volume over 0.1 s (FEV0.1) and forced vital capacity (FVC), which were used to determine the ratio between FEV0.1/FVC as a clinical measure of lung performance. Three independent measurements were taken for all perturbations for each mouse and the average was calculated.

### Eye Histology

Paraffin-embedded eyes were sectioned at 3 μm thickness and every 20th section was stained with H&E. Four photomicrographs (x10) of each cross-section were captured across the full circumference of the eye and the average diameter (in μm) per section for each eye was determined to obtain choroidal thickness as previously described (17). Investigators were masked to the experimental groups.

### Statistical Analysis

Values are presented as median ± IQR. Results from individual models were compared to the room air controls using the non-parametric unpaired *T*-test (Mann-Whitney) in GraphPad Prism software (version 4.03, SD, USA); *P* < 0.05 was considered statistically significant. Unmarked bars on figures indicate that no significance was achieved.

## Results

### Body Weight Gain Is Affected in Mice Exposed to High Oxygen for Longer Periods

To define the best experimental model that most closely recapitulates BPD, we trialed three oxygen exposure protocols (Supplementary Figures 1A–C). For each of these, we used a moderate oxygen concentration of 75%, as we have previously reported that this concentration is sufficient to elicit both early and long-term damage in the neonatal lung and eye (17). Oxygen exposure in neonatal pups for 5 days had no effect on body weight gain at PN14 or PN40 as their weight was comparable to that of age-matched room air control mice (Supplementary Figures 1D,E). Conversely, neonatal mouse pups exposed to oxygen for 8 days had reduced body weight gain at PN14, which had normalized by PN40 (Supplementary Figures 1D,E). However, mice exposed to high oxygen for 14 days had reduced body weight gain at both PN14 and PN40 compared to mice exposed to high oxygen for shorter periods and age-matched room air controls, suggesting a more profound impact (Supplementary Figures 1D,E).

### Prolonged Oxygen Exposure Leads to BPD-Like Damage in Neonatal Mice

**Figure 1:**
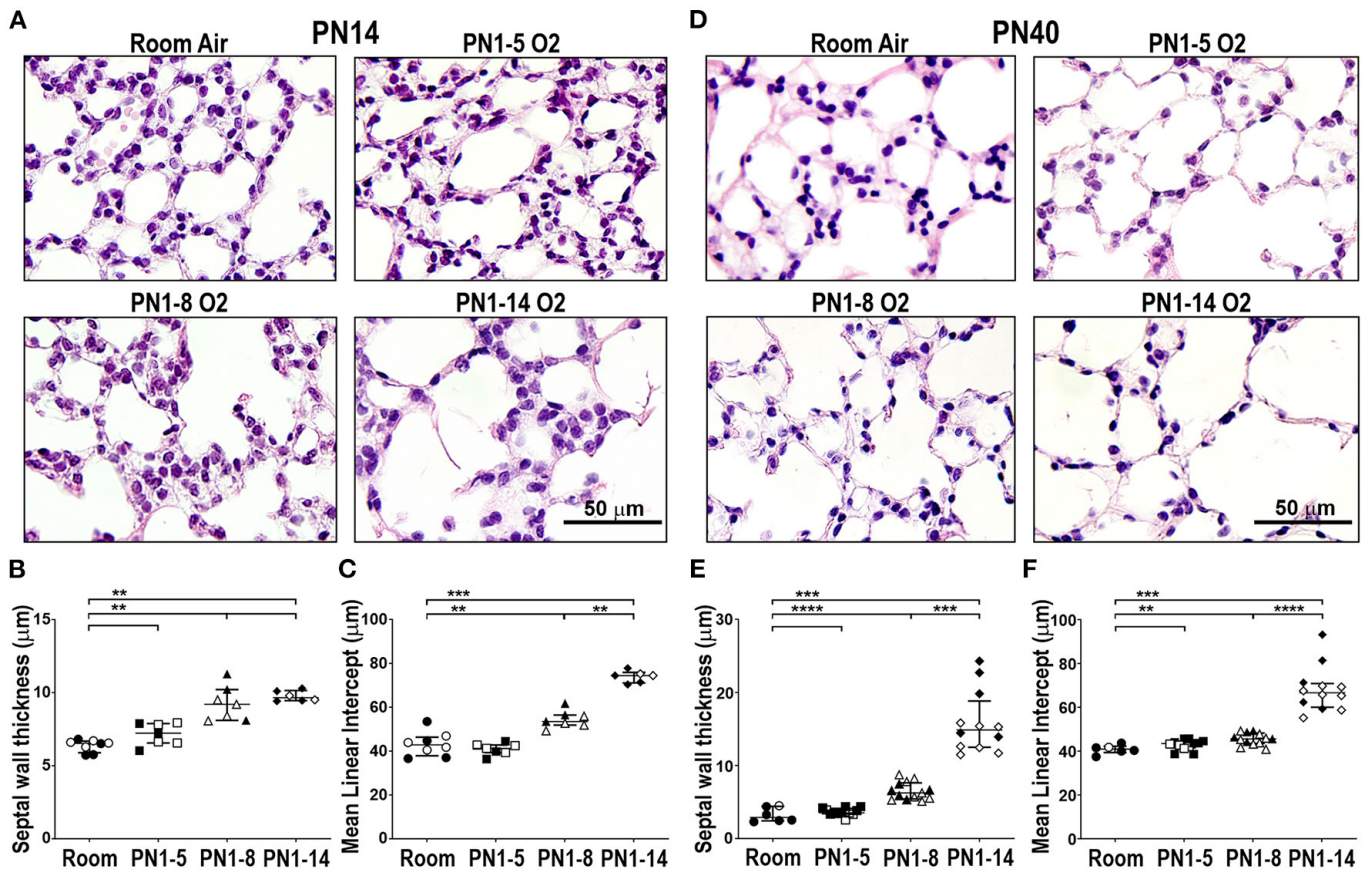
Prolonged supplemental oxygen induces severe lung damage in neonatal mice that persists into young adulthood. C57BL/6 mice were housed in room air or treated from the day of birth with 75% O_2_ for 5, 8, or 14 days, then analyzed at PN14 **(A–C)** or PN40 **(D–F)**. **(A)** Representative photomicrographs of H&E-stained lung cross-sections at PN14. **(B)** Median alveolar septal wall thickness of mice in **(A)**. **(C)** Alveolar airspace size by mean linear intercept length (μm) of mice in **(A)**. **(D)** Representative photomicrographs of H&E-stained lung cross-sections at PN40. **(E)** Median alveolar septal wall thickness of mice in **(D)**. **(F)** Alveolar airspace size by mean linear intercept length (μm) of mice in **(D)**. Images in **(A)** and **(D)** were taken using a 40x objective using an Olympus BX-51 bright field microscope. Scale bar = 50 μm. For **(B,C,E,F)**, data is median (μm) ± IQR. **P < 0.01, ***P < 0.001, ****P < 0.0001 by Mann-Whitney *U*-test (2-tailed). n ≥ 6 mice per group, with 1–2 L used to analyze each oxygen group in **(A–C)**, and 2–3 L used to analyze each oxygen group in **(D–F)**. Gender is represented by closed (male) and open symbols (female). PN, postnatal.

In mice, the bulk of alveolarization in the lung is complete by PN14 (28), forming a suitable time-point for assessment of the major structural changes to the alveoli. We used two primary measurements, alveolar septal wall thickness and airspace size, to evaluate the structural damage induced in oxygen-exposed mouse lungs. Mice reared in room air from the day of birth, up to and including PN14, had normal alveolar structure, as shown by typical alveolar septal wall thickness and airspace diameter (Figures 1A–C). Exposure to 75% oxygen for the first 5 days of life (PN1-5) had minimal effect on the visual appearance of the lung. Morphometric measurement revealed a possible thickening of the septal wall compared to room air controls; however, this was not significant and there was no difference in airspace size between the oxygen-exposed and room air groups (Figures 1A–C). Extending the duration of oxygen exposure to 8 days (PN1-8) induced a change in parenchymal architecture, demonstrated by an increase in alveolar septal wall thickening and alveolar diameter (Figures 1A–C). Lengthening the oxygen exposure further to 14 days (PN1-14) gave rise to lungs with severe alveolar deformities, showing a marked increase in septal wall thickening and exaggerated alveolar size compared to room air controls. The airspace diameter in this group showed even greater enlargement compared to mice exposed to oxygen for 8 days (Figures 1A–C).

### Prolonged Neonatal Oxygen Exposure Leads to the Development of Emphysema in Young Adult Mice

It is now well-appreciated that preterm infants diagnosed with BPD have an increased susceptibility to other lung diseases in later life, including COPD (6, 7). To examine the longer-term impact of neonatal oxygen exposure, the lungs of mice administered 75% oxygen for the first 5, 8, or 14 days of life, were examined at PN40. Mice that had been exposed from birth to 75% oxygen for 8 days (PN1-8), but not 5 days (PN1-5), showed modest yet significant increases in septal wall thickness and airspace size compared to age-matched room air controls at PN40 (Figures 1D–F). Interestingly, in the PN1-8 model, both the septal wall thickness and airspace size at PN40 were somewhat reduced compared to measurements at PN14, suggesting possible healing (Figures 1C–F). However, mice that had been exposed for their first 14 days of life to 75% oxygen demonstrated pronounced structural alternations in lung parenchyma, including a variable but highly significant increase in septal wall thickness and airspace diameter compared to mice exposed to oxygen for 8 days (Figures 1D–F). This finding demonstrates the capacity of the PN1-14 oxygen model to generate structural changes consistent with emphysema in adulthood (29).

### Oxygen Exposure Did Not Induce Severe Parenchymal Fibrosis in Neonatal and Adult Lung

**Figure 2:**
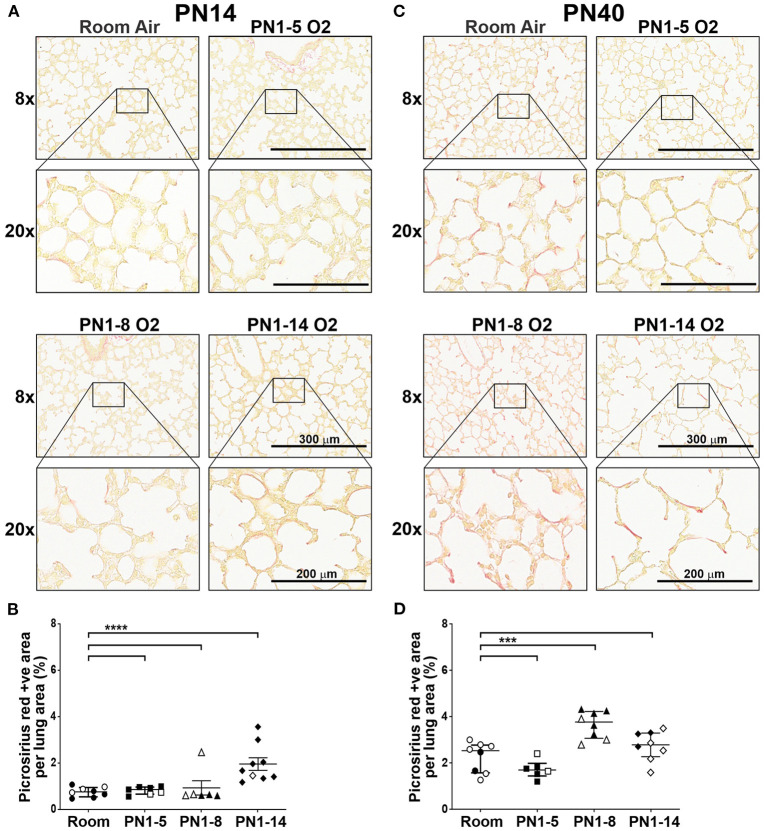
Extensive fibrosis is not a feature of the lungs of neonatal or young adult mice exposed to prolonged supplemental oxygen in early life. C57BL/6 mice were housed in room air or treated from the day of birth with 75% O_2_ for 5, 8, or 14 days, then analyzed at PN14 **(A,B)** or PN40 **(C,D)**. **(A)** Representative images of PR-stained cross-sections of lungs at PN14. **(B)** Proportion of lung tissue of the mice in **(A)** stained with PR. **(C)** Representative images of PR-stained cross-sections of lungs at PN40. **(D)** Proportion of lung tissue of the mice in **(C)** stained with PR. For **(A,C)**, images were taken with 8x and 20x objectives using Aperio ScanScope CS; scale bar = 300 and 200 μm, respectively. For **(B,D)**, data is presented as median (μm) ± IQR. ***P < 0.001, ****P < 0.0001 by Mann-Whitney *U*-test (2-tailed). *n* ≥ 6 mice per group, with 2–3 L used to analyze each oxygen group in **(A,B)**, and 1–3 L used to analyze each oxygen group in **(C,D)**. Gender is represented by closed (male) and open symbols (female). PN, postnatal.

A phenotype of “old” BPD is the presence of extensive parenchymal fibrosis, however this trait is significantly milder in the lungs of infants with newly diagnosed BPD due to the changes in respiratory care implemented in the NICU over the last few decades (3, 30). Thus, contemporary experimental models should not feature severe parenchymal fibrosis. To determine the prevalence of fibrosis, the lungs of mice at PN14 were stained with Picrosirius Red (PR), a histological stain that renders collagen fibers red. Minimal collagen staining was observed in the extracellular matrix of the lungs of all mice at PN14, except mice exposed to 75% oxygen for 14 days (Figures 2A,B). While there was a significant increase in parenchymal fibrosis in mice exposed to high oxygen for 14 days, on average, only 1.94% of the lung tissue in this model was positive for PR (Figures 2A,B). When fibrosis was assessed at day 40, only mice exposed to 8 days of high oxygen demonstrated a significant increase in the proportion of lung tissue positive for PR compared to room air control mice, although changes were mild (Figures 2C,D). Thus, the concentration and window of oxygen exposure did not lead to the development of extensive fibrosis resembling old BPD in any of the three models tested in neonatal and adult life.

### Eight Days of Oxygen Exposure in Neonatal Mice Stimulates Mild Expansion of Type II Alveolar Epithelial Cells in Adulthood

**Figure 3:**
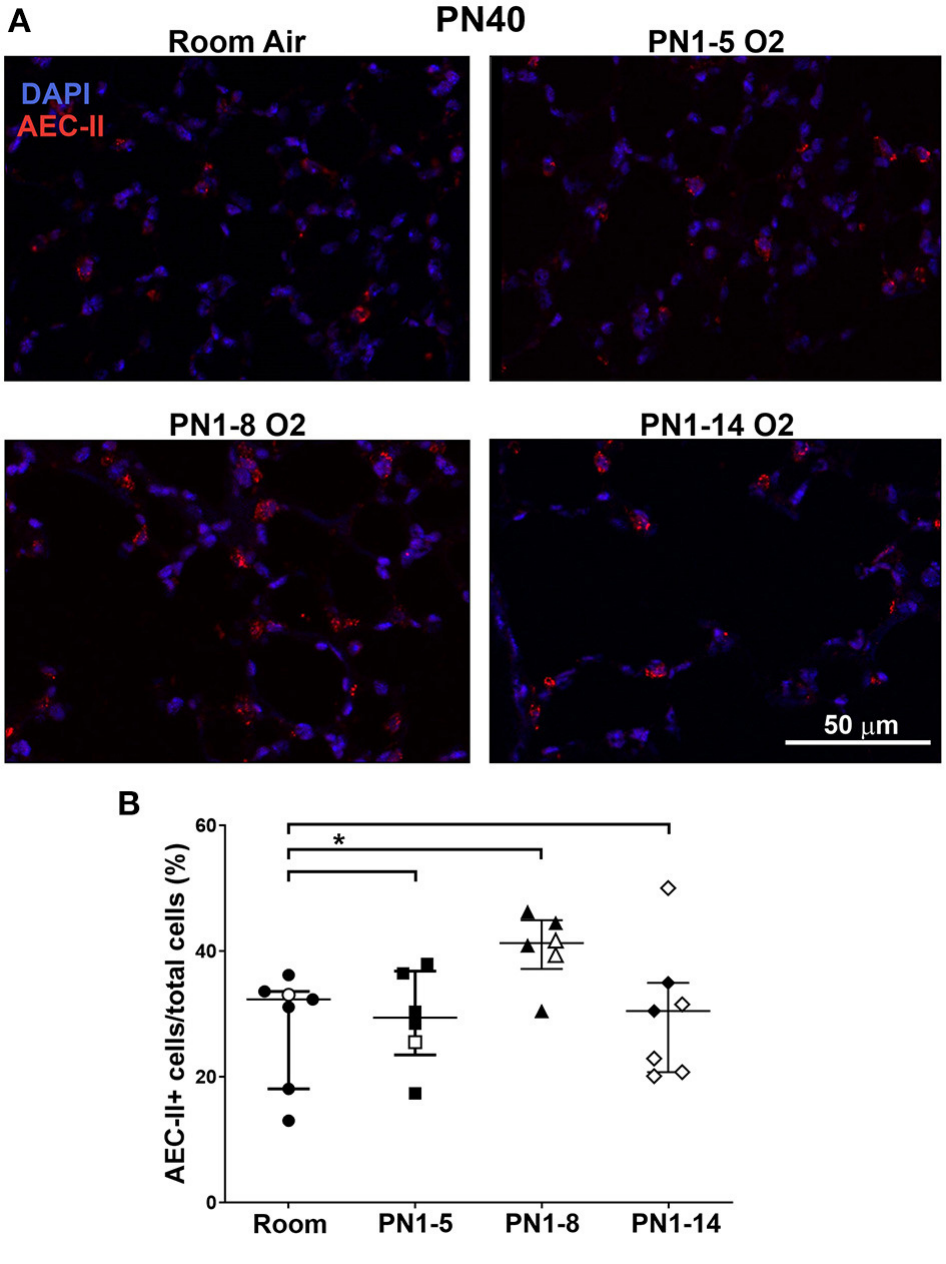
Neonatal oxygen promotes mild AEC-II expansion in adulthood. **(A)** Representative immunofluorescent images of lungs from room air control mice or mice exposed to 75% oxygen for the indicated times. Staining was undertaken at PN40 with anti-Pro-SPC (red) to depict AEC-II. DAPI-stained nuclei appear blue. Scale bar = 50 μm. Images were taken with 20x glycerol objective using a Nikon A1r inverted confocal microscope, zoom setting 2. **(B)** Quantitation of proportions of AEC-II+ cells indicated as AEC-II+/total lung cells. Data is presented as median ± IQR. *P < 0.05 by Mann-Whitney *U*-test (2-tailed). *n* ≥ 6 mice per group, with 2–4 L used in the analysis of each oxygen group. Gender is represented by closed (male) and open symbols (female). PN, postnatal.

Hyperoxia has been associated with impaired AEC development, promoting an enlarged and simplified lung structure that is commonly observed in BPD (26, 31). To determine if the high oxygen insult utilized in the three different models induced changes in the proportions of type II AECs in the lung parenchyma of adult mice, immunostaining of Pro-SPC was performed. At PN40, proportions of AEC-II in the lungs of mice exposed to high oxygen for 5 or 14 days were comparable with those in room air control mice, however surprisingly, there was a mild expansion of AEC-II in the lungs of mice exposed to 75% oxygen for 8 days (Figures 3A,B).

### Neonatal Oxygen Exposure Induces Alveolar Inflammation in Early BPD

**Figure 4:**
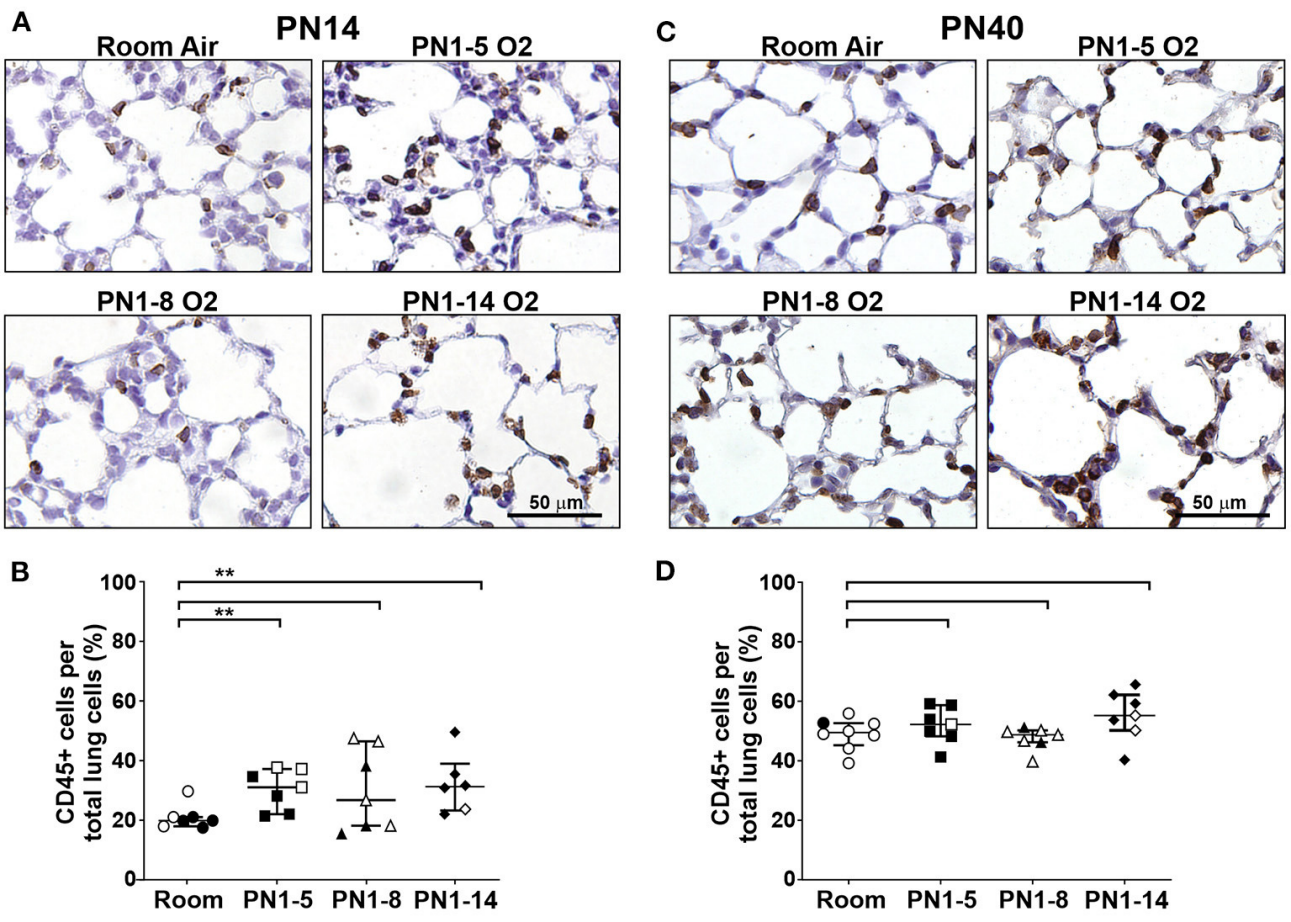
Inflammation is present in the lungs of neonatal mice exposed at birth to supplemental oxygen but is moderated in young adult mice. C57BL/6 mice were housed in room air or treated from the day of birth with 75% O_2_ for 5, 8, or 14 days, then their lungs were analyzed at PN14 **(A,B)** or PN40 **(C,D)** by staining with the pan-leukocyte cell surface marker CD45. **(A)** Representative images of lung paraffin sections stained at PN14, and **(B)** quantitation of the proportion of CD45+ immune cells per total alveolar cells. **(C)** Representative images of lung paraffin sections stained at PN40, and **(D)** quantitation of the proportion of CD45+ immune cells per total alveolar cells. In **(A,C)**, sections were counter-stained with hematoxylin and CD45+ cells are indicated by brown staining; scale bar = 50 μm. Note that lungs were not perfused or flushed prior to fixation and extraction. Images were taken with 40x objective using an Olympus BX-51 bright field microscope. Data in **(B,D)** is presented as median ± IQR. **P < 0.01 by Mann-Whitney *U*-test (2-tailed). *n* ≥ 6 mice per group, with 1–2 L used for analysis of each oxygen group in **(A,B)**, and 2–3 L used for analysis of each oxygen group in **(C,D)**. Gender is represented by closed (male) and open symbols (female). PN, postnatal.

In order to investigate the effect of high oxygen exposure on lung inflammation in the neonate, immunohistochemical staining for CD45-expressing leukocytes was performed. In the normal developing lung at PN14, ~20% of the cells present were CD45+ leukocytes, which were mostly found within the alveolar walls, around small vessels and occasionally also in the airspaces (Figures 4A,B). In all high oxygen-exposed models there was a variable increase in leukocytes in the lung, ~10–15% more than in the room air control mice, which was significant in the PN1-5 and PN1-14 models (Figures 4A,B). Thus, an increase in lung inflammation is a feature of high oxygen exposure of neonatal mice.

### Alveolar Inflammation Is Not a Feature of Adult Mice Exposed to an Oxygen Insult in Infancy

To determine if the high oxygen insult utilized in the three different models led to sustained inflammation in the lung parenchyma, immunostaining was performed on lung sections from 40-day-old mice. At PN40, proportions of CD45+ leukocytes in all high oxygen exposure models were comparable with the room air control mice (Figures 4C,D) indicating that lung inflammation observed at the early time period had resolved by adulthood.

### Prolonged Oxygen Exposure Leads to Choroidal Thinning in Adulthood

**Figure 5:**
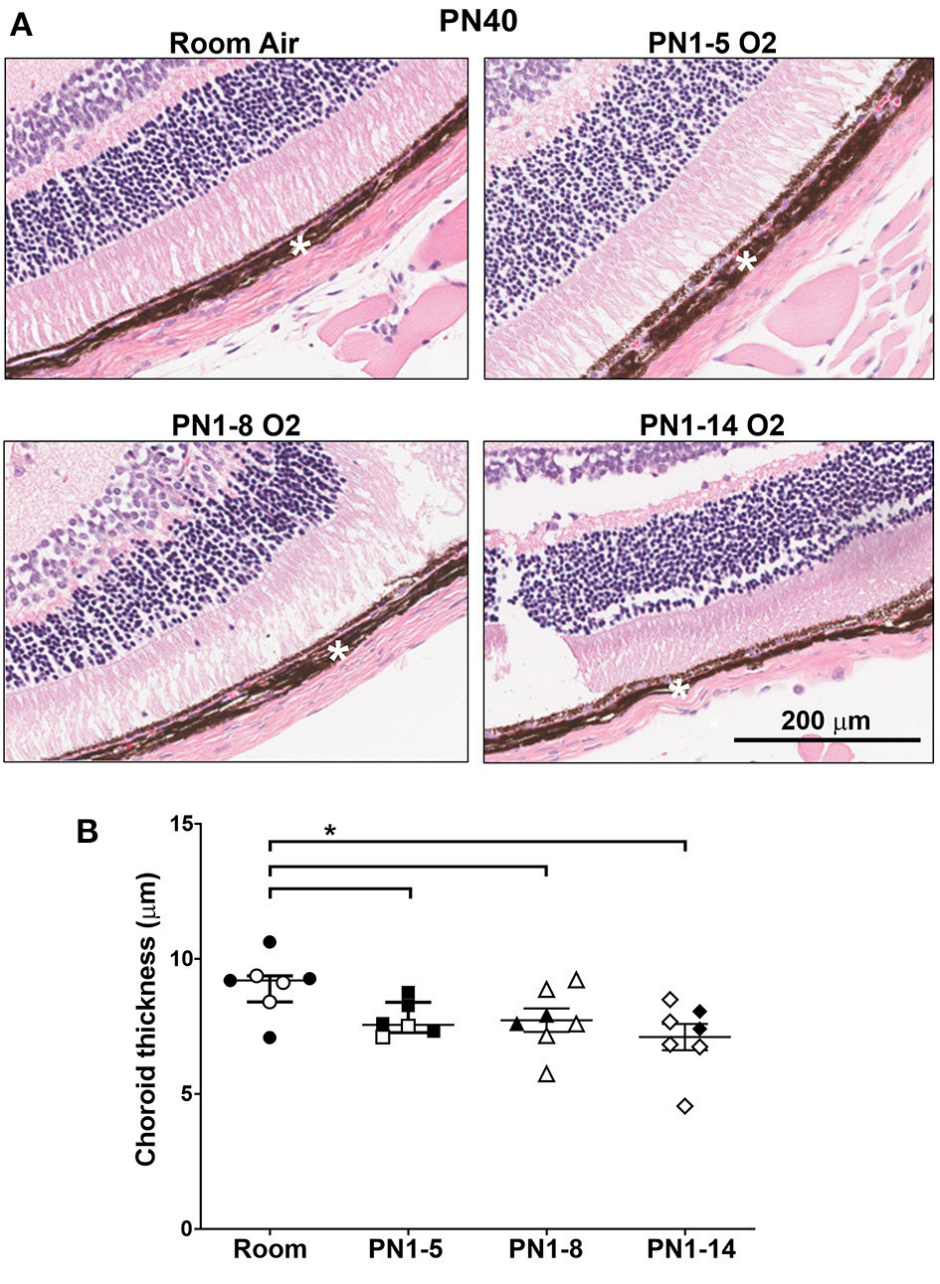
Choroidal thinning in young adult mice exposed to prolonged oxygen in infancy. **(A)** Representative H&E photomicrographs of sections depicting choroid (white asterisk) at PN40 comparing C57BL/6 room air control mice and the indicated oxygen-exposed mice. Images were taken with 10x objective using Aperio ScanScope CS. Scale bar = 200 μm. **(B)** Quantitation of choroidal thickness (asterisk) of eye sections shown in **(A)**. All data are presented as median ± IQR and assessed using Mann-Whitney *U*-test (2-tailed); *P < 0.05. *n* ≥ 6 mice per group, with 2 L used in the analysis of each oxygen group. Gender is represented by closed (male) and open symbols (female). PN, postnatal. The retinas were obtained from the same mice used to assess lung pathology in other figures. Outliers were removed using a ROUTS outlier test.

The neonatal retina is highly susceptible to changes in oxygen tensions and it is a prominent co-morbidity of BPD. Survivors of this eye condition exhibit thinning of the outer retina (choroid). To evaluate the impact of neonatal high oxygen exposure on the thickness of the outer retina, H&E-stained eye sections from 40-day-old mice were examined. At PN40, there was a trending decrease in choroidal thickness in mice exposed to high oxygen for 5 (*p* = 0.0513) and 8 days (*p* = 0.0728) but it was significantly decreased in mice exposed to high oxygen for 14 days compared to room air control mice (Figures 5A,B). These results indicate that extended oxygen exposure in early infancy was sufficient to induce concurrent outer retinal changes in adulthood.

### Lung Function Is Impaired in Mature Adult Mice Exposed to Hyperoxia in Infancy

**Figure 6:**
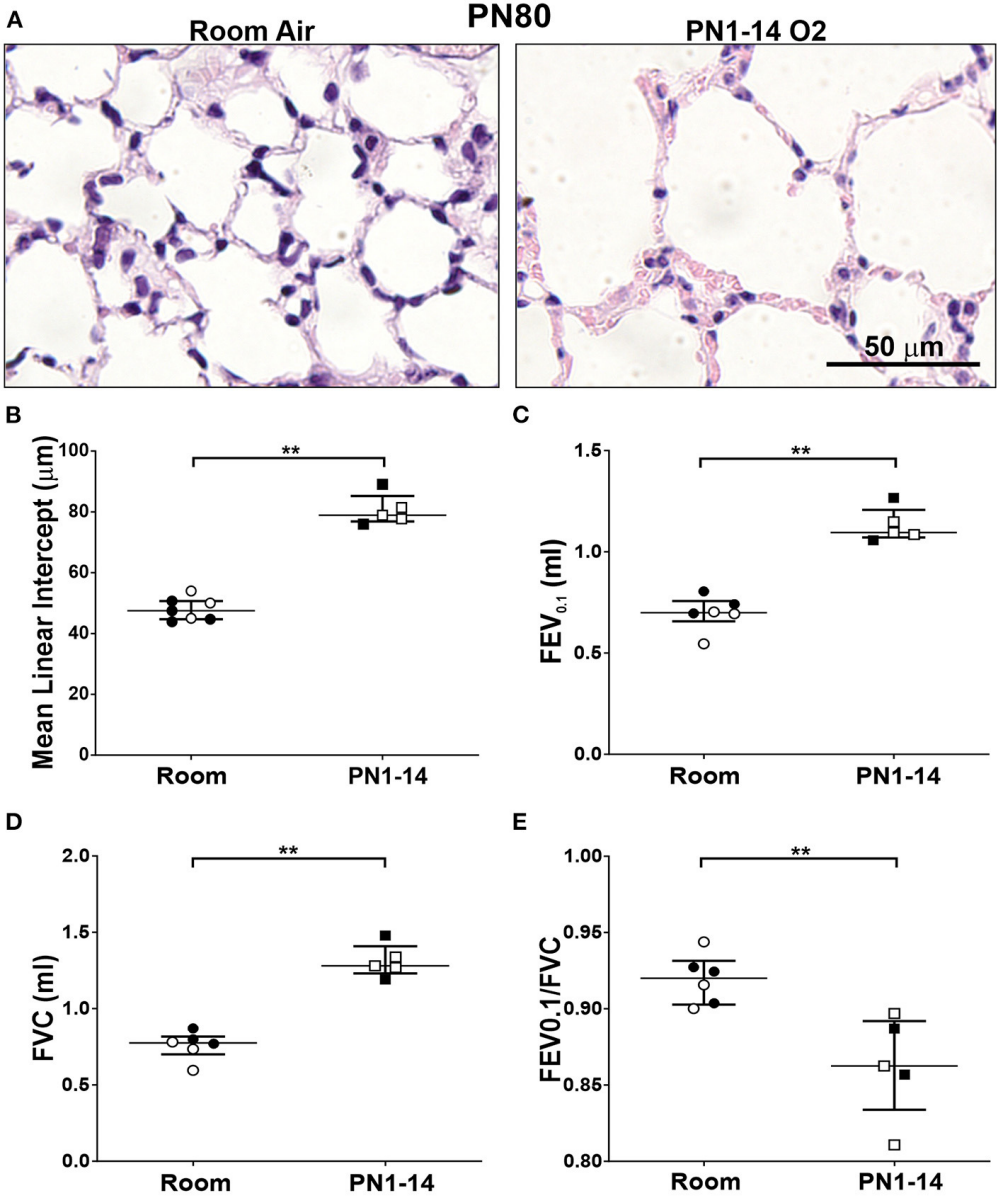
Decline in lung function in mature adult mice exposed to prolonged supplemental oxygen in infancy. **(A)** Representative photomicrographs of H&E-stained lung cross-sections at PN80 comparing C57BL/6 room air control mice and PN1-14 oxygen-exposed mice. Images were taken with a 40x objective using an Olympus BX-51 bright field microscope. Scale bar = 50 μm. **(B)** Quantitation of airspace diameter (MLI) of lung sections shown in **(A)**. Assessment of **(C)** FEV0.1, **(D)** FVC, and **(E)** FEV0.1/FVC ratio by negative pressure-driven forced expiration using the Flexivent. All data are presented as median ± IQR and assessed using Mann-Whitney *U*-test (2-tailed); **P < 0.01. *n* = 5–6 mice per group from 1 litter. Gender is represented by closed (male) and open symbols (female). PN, postnatal.

A vital question arising from the above data is whether the structural deterioration observed in mice exposed to prolonged hyperoxia worsens structurally and functionally with age. At PN80, mature adult C57BL/6 mice that had been exposed from day of birth to 14 days of hyperoxia showed progressive alveolar hypoplasia, with larger and simplified alveoli, as indicated by increase in alveolar diameter, compared to those reared solely in room air conditions (Figures 6A,B). In patients with COPD, persistent airflow limitation is a clinical indicator of disease progression (32, 33). A significant increase in FEV0.1 and FVC was observed at PN80 in C57BL/6 mice that had received an oxygen insult during the first 14 days of neonatal development compared to room air control counterparts (Figures 6C,D). The ratio between FEV0.1/FVC, which is a clinical measure of COPD in humans at FEV at 1 s, was significantly decreased (< 0.9) in oxygen exposed mice at PN80 (Figure 6E). These findings indicate that prolonged oxygen exposure in early infancy that induces BPD can progress into pulmonary function decline resembling COPD in adulthood.

## Discussion

There is an unresolved need for effective preventative and reparative treatments that can overcome the long-term respiratory burden faced by preterm infants that develop BPD. Over many years, the use of animal models has proven to be valuable for uncovering new disease mechanisms and new targets for therapy in a variety of human diseases. In BPD, a standardized experimental model is not used routinely and instead, many studies have used varying concentrations of oxygen, different lengths of oxygen exposures, contrasting initiation times, and additionally, insults to the mouse dam to promote an inflammatory environment, which only occurs in a small proportion of very low birth weight infants. Here, we investigated the long-term effect of 75% oxygen delivered at various developmental windows of lung organogenesis in the neonate, to best replicate the timing of injury in premature infants at risk of developing BPD and to assess the long-term repercussions of this early-life oxygen exposure on the lung but also to ocular structures such as the choroid. Importantly, all three models evaluated were initiated within 12 h of birth of the mouse.

Our findings show that two of the three models tested induced damage to the alveolar compartment in neonatal and adult mice, increasing in severity as the window of oxygen exposure lengthened. While both the PN1-8 and PN1-14 models yielded lung disease characteristics common to infants affected by BPD, the PN1-14 model yielded more exaggerated lung disease traits, particularly alveolar airspace enlargement at PN14 and PN40. At PN80, the PN1-14 model led to worsened emphysema, which was associated with a decline in respiratory mechanics, an important characteristic that is often overlooked. Therefore, our research supports that the delivery of 75% oxygen to mouse pups for 14 days is the optimal model for standardized use in BPD research, to investigate the early and long-term effects of neonatal hyperoxia.

In other hyperoxia models, prolonged exposure to high oxygen (85–100%) for 14 days or longer has been reported to result in the development of extensive fibrosis in the lung parenchyma (21, 34–36). This feature was prevalent in the early form of human BPD, that was encountered in the past (21). However, since the introduction of surfactant therapy, antenatal corticosteroids and advanced neonatal oxygen management, the BPD phenotype observed today no longer features severe pulmonary fibrosis (37). Thus, to accurately model contemporary BPD, it is important to understand whether mice delivered a high oxygen insult develop excessive parenchymal fibrosis, which is a feature that is not regularly considered in other studies. Despite a slight but significant increase in PR staining in the lung of mice exposed to high oxygen for 14 days, none of the models exhibited marked fibrosis in the extracellular matrix of the alveoli at the 14-day assessment timepoint. Interestingly, all mice showed mild increases in fibrosis between day 14 and 40, which likely reflects developmental remodeling of the extracellular matrix during lung alveolarization. However, mice that had been exposed to high oxygen for the first 8 days of life exhibited a significant increase in lung fibrosis at day 40 compared to room air controls that was not seen in the other models. While the reason for this is unknown, it is interesting that these mice were also the only group to show a significant increase in AEC-II numbers at day 40. This collectively implies a healing response, which is also suggested by a reduction in the thickness of septal walls and airspace size at PN40 compared to that seen at PN14. These features were not seen in mice given a prolonged oxygen insult, which may suggest that their lungs have become too damaged. Thus, we confirm that it is suitable to use a high oxygen concentration of 75% for up to 14 days in C57BL/6 mice to emulate the form of BPD that prevails in the neonatal clinic today.

Targeting oxygen exposure within the critical window of lung development in experimental models is an important consideration to maintain clinical relevance to human BPD. Importantly, the saccular and alveolar stages of lung development appear to be most impacted during oxygen administration in preterm infants in NICUs (38). The mouse represents an excellent system to model BPD, as the saccular stage of lung development, which is generally complete in full-term infants, occurs predominantly in the first 5 days of postnatal life of the mouse. In the first model tested, oxygen exposure was limited to this important developmental stage. This scheme did not induce major structural changes in the neonatal or adult lung, despite a hint of septal wall thickening at day 14. Previous studies have reported that this short window of exposure during the saccular stage of lung development was sufficient to elicit severe alveolar damage in 8–9-week-old adult mice (13, 39). This difference likely relates to the lower concentration of oxygen used in our model but may also be due to the housing environment or the assessment timepoint, since we examined mice at 40 days of age (13, 39). Thus, it is possible that lung defects may manifest later on if the damage progresses at a slower rate. In a study exposing neonatal pups to 40, 60, and 100% oxygen from PN1-4, only 100% oxygen led to lung dysmorphogenesis in C57BL/6 mice; however, outbred CD1 mice exhibited lung development changes in response to 60% oxygen as well (40), indicating that genetic background contributes to the response of the lungs to an oxygen insult. In the second model we tested, mice were exposed to high oxygen for 8 days, which encompasses the saccular stage and the start of the alveolar phase of lung development. Interestingly, this slightly longer insult led to the development of enlarged and simplified airspaces at both assessment timepoints, albeit lung damage at PN40 was moderate. There was also an expansion of AEC-II cells observed in the lungs of this model at PN40, which was not apparent in the PN1-5 and PN1-14 protocols at this timepoint. This was an unexpected finding as fewer AEC-II cells have previously been observed in adult mice recovering from neonatal oxygen exposure (41). However, in that study, newborn mice were exposed to 100% oxygen, which is a severe oxygen insult compared to the 75% oxygen concentration used in this study and one that is unlikely to be utilized in the NICU. Nevertheless, oxygen concentration and timing could influence the degree of activation/inhibition of different cell populations in the lung (41).

As expected, 14 days of high oxygen exposure generated the greatest degree of alveolar dysmorphogenesis in the neonatal lung, which manifested as severe pulmonary emphysema in the young and mature adult. The impact of this structural deterioration on lung mechanics was evident by the impaired respiratory function in mature adults exposed to this oxygen scheme. Taken together, these findings indicate that prolonged high oxygen exposure during the two final lung developmental stages in mice promotes lung injury that approximates that seen in preterm infants with BPD, and may even capture those infants that go onto to develop COPD in later life. This model will therefore also be useful to unravel mechanisms that underpin chronic lung conditions that have origins in early life. The ability of the 14-day exposure regime to generate long-term lung damage and functional decline within a relatively short time frame compared to current models in use (42, 43), also provides a unique advantage when trialing potential therapeutic interventions for BPD. For example, the efficacy of a select intervention to ameliorate or attenuate long-term damage can be determined in mice within 40 days of birth, making testing of therapeutics easily achievable. Another important consideration is the emerging influence of the lung and gut microbiome on the development of BPD. Given that selective pressures such as moisture, pH and nutrition can shape the local microbial niche (44), mice housed in different animal facilities are subsequently exposed to diverse environmental conditions. Therefore, these factors may also influence the severity of lung pathology generated in oxygen-exposed mice and could also account for the discrepancies observed between different research groups using the same oxygen models.

Inflammation is also recognized to contribute to the deterioration of the alveolar structure in the neonatal lung following a high oxygen insult (45). Airway sections obtained from preterm infants with BPD often show elevated levels of pro-inflammatory chemokines and cytokines, and can occur early in neonatal life before the presentation of clinical symptoms (46, 47). However, infants with BPD have demonstrated increased susceptibility to COPD, respiratory viral infections and asthma in later life compared to their non-BPD counterparts, which may involve the dysregulation of immune pathways in the lung during neonatal hyperoxia exposure (13). In a previous study, exposure to hyperoxia during the first 12 days of neonatal life led to persistent inflammation in the lungs of adult mice, implicating chronic inflammation in the lung of BPD survivors (48). To determine whether oxygen exposure at varying developmental windows would influence the immune profile in the adult lung, immunohistochemical staining for CD45+ leukocytes was conducted on lung tissue. All mice showed an increase in leukocytes in lung between day 14 and 40, which likely reflects the maturation of the immune system, which undergoes rapid development during the early postnatal period; however, alveolar inflammation at day 40 was not a feature of any of the three high oxygen regimes trialed in this study. This suggests that the structural changes apparent in adulthood are most likely the consequences of oxygen exposure during critical lung stages in infancy (49). Concurrently, there may also be changes occurring in other tissue components external to inflammation, such as modifications to extracellular matrix proteins (50).

A common clinical co-morbidity of infants diagnosed with BPD is ROP (51). The retinal vasculature is sensitive to the changes in oxygen tension and extreme hyperoxia/hypoxia can hinder the normal growth of the blood vessels in the retina which can lead to poor vision in later life (52). Adult survivors of ROP have been reported to exhibit late-onset vitreoretinal complications (53) and retinal detachment (54). In addition, a thinner choroid has been associated with reduced vision in individuals with a history of ROP, and has been speculated to be primary to the long-term changes in the inner retinal layers (55). Therefore, the degree of choroidal thinning was assessed across all three oxygen models and presents a unique aspect of this study. While only the 14-day exposure model showed a significant reduction in choroidal thickness, the other two models displayed trending degrees of vascular thinning. This demonstrates that an extended period of high oxygen exposure in neonatal life can also provoke long-term damage in organs outside of the lung and evaluation of other tissues implicated in BPD could enhance the clinical relevance and translatability of this model. However, the timing of the oxygen exposure soon after birth in the 14-day model employed in this study is considerably different to that used in the established model of ROP, known as the oxygen-induced retinopathy model. Due to differences in retinal development between mouse and man, high oxygen exposure in the oxygen-induced retinopathy model commences on postnatal day seven to mimic the vascular developmental stage in preterm infants (56). Therefore, the specific molecular changes underlying the vascular damage in the retina may be grossly different between the two oxygen schemes and should be considered closely before use in ocular research.

In the absence of a cure for BPD, there is a need to identify the mechanisms underlying this debilitating condition. Accordingly, the development of a benchmark model of BPD will allow for greater comparison of results amongst research groups and advances in the understanding of the disease. The current study describes a model of oxygen-induced BPD, comprised of a 14-day exposure to 75% oxygen, initiated within 12 h of birth, which was associated with features of contemporary BPD in neonatal mice. Moreover, the model was associated with the impaired development of alveoli in adult mice that resembled COPD encountered by adult survivors of BPD alongside outer retinal decay, which is a distinguishing feature of this model. These findings provide a distinct advantage, suggesting that this model could be a key translational tool for the trialing of new treatments which may advance therapeutic strategies to improve the care and long-term outcome for vulnerable premature infants. We advocate that the standardized PN1-14 model described herein may facilitate studies into the early life impact of high oxygen-exposure but also expedite investigations into the lifelong effect of oxygen insults received during the neonatal period.

## Data Availability Statement

The raw data supporting the conclusions of this article will be made available by the authors, without undue reservation.

## Ethics Statement

The animal study was reviewed and approved by Alfred Research Alliance Animal Ethics Committee (experimental approval number: E/1746/2017/M).

## Author Contributions

MH and LW conceived the study, designed research, and analyzed data. LW performed experiments. PW supplied an integral piece of equipment, provided critical intellectual content, and important manuscript revisions. CJ contributed a new analytic tool. ET provided valuable insight and made important intellectual contributions. LW and MH wrote the paper. All authors provided editorial comments and gave final approval for publication.

## Conflict of Interest

The authors declare that the research was conducted in the absence of any commercial or financial relationships that could be construed as a potential conflict of interest.

## Publisher's Note

All claims expressed in this article are solely those of the authors and do not necessarily represent those of their affiliated organizations, or those of the publisher, the editors and the reviewers. Any product that may be evaluated in this article, or claim that may be made by its manufacturer, is not guaranteed or endorsed by the publisher.

## Supplementary Material

Supplementary Figure 1 Oxygen exposure models trialed in this study. Within 12 h of birth, neonatal C57BL/6 mice were exposed to **(A)** 75% O2 for 5 [PN1-5], **(B)** 8 [PN1-8], or **(C)** 14 days [PN1-14] with a daily 3-h period in room air (not shown in figure). Cohorts of mice were analyzed at PN14 or PN40, and for the PN1-14 model at PN80 (red arrows) alongside room air control mice. Body weights of room air control mice and oxygen-exposed mice at **(D)** PN14 or **(E)** PN40. ^**^*P* < 0.01 and ^***^*P* < 0.001 by Mann-Whitney *U*-test (2-tailed). *n* ≥ 6 mice per group, with 1–2 L used in the analysis of each oxygen group. Gender is represented by closed symbols (male) and open symbols (female). PN, postnatal.

Supplementary Table 1 Custom imageJ script used for the quantitation of fluorescence positive cells.
