# Supplementary figures and images for: An Experimental Model of Bronchopulmonary Dysplasia Features Long-Term Retinal and Pulmonary Defects but Not Sustained Lung Inflammation
Source: Front Pediatr. 2021 Aug 30;9:689699. doi: 10.3389/fped.2021.689699 (PMC8435611; doi:10.3389/fped.2021.689699)

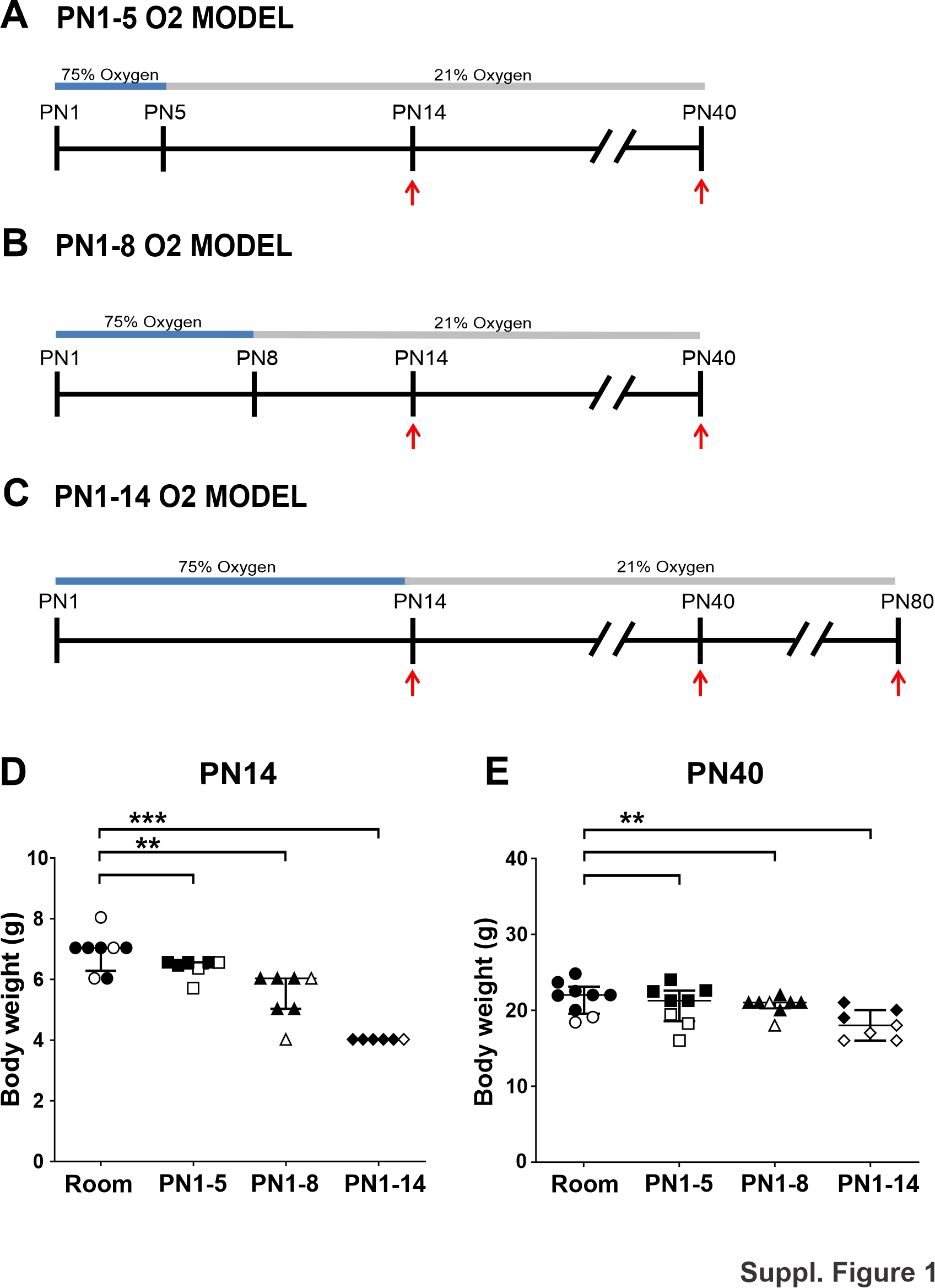

Supplement: Supplementary Figure 1 — Oxygen exposure models trialed in this study. Within 12 h of birth, neonatal C57BL/6 mice were exposed to (A) 75% O2 for 5 [PN1-5], (B) 8 [PN1-8], or (C) 14 days [PN1-14] with a daily 3-h period in room air (not shown in figure). Cohorts of mice were analyzed at PN14 or PN40, and for the PN1-14 model at PN80 (red arrows) alongside room air control mice. Body weights of room air control mice and oxygen-exposed mice at (D) PN14 or (E) PN40. **P < 0.01 and ***P < 0.001 by Mann-Whitney U-test (2-tailed). n ≥ 6 mice per group, with 1–2 L used in the analysis of each oxygen group. Gender is represented by closed symbols (male) and open symbols (female). PN, postnatal. [file Image_1.JPEG]
