# Supplementary material for: An Experimental Model of Bronchopulmonary Dysplasia Features Long-Term Retinal and Pulmonary Defects but Not Sustained Lung Inflammation
Source: Front Pediatr. 2021 Aug 30;9:689699. doi: 10.3389/fped.2021.689699 (PMC8435611; doi:10.3389/fped.2021.689699)
Supplement: Supplementary Table 1 — Custom imageJ script used for the quantitation of fluorescence positive cells. [file Table_1.DOCX]

**Supplemental Table S1 Custom ImageJ script used for the quantitation of Fluorescence Positive Cells**

var Nucleus;

var Analysis1;

var Analysis2;

var CellMask;

var Maxima = 900;

var Sensitivity1 = 1500;

var Sensitivity2 = 1500;

var ROICOUNT = 0;

var TotalCells = 0;

var GPositive = 0;

var GNegative = 0;

var GPercPos = 0;

var RPositive = 0;

var RNegative = 0;

var RPercPos = 0;

var GRPositive = 0;

var GRNegative = 0;

var GRPercPos = 0;

var GCellsPerArea;

var RCellsPerArea;

var GRCellsPerArea;

var TissueArea;

var Scale = 100;

var lower;

var upper;

var lower2;

var upper2;

var lower3;

var upper3;

var ThreshTrigger = 0;

var ThreshTrigger2 = 0;

var ThreshTrigger3 = 0;

var Output1Mask;

var Output2Mask;

var Vignette;

var VignetteCounter = 0;

//Show dialog to get Sample Information

roiManager("reset");

run("Close All");

run("Set Measurements...", "area mean min limit display redirect=None decimal=3");

Dialog.create("Dual Channel Fluorescence Analysis");

items1 = newArray("1","2","3","4");

items2 = newArray(".nd2",".tif");

items3 = newArray("Manual Thresholding", "Maximum Intensity");

items4 = newArray("Yes", "No");

Dialog.addRadioButtonGroup("Select Nucleus Channel", items1, 1, 3, "1");

Dialog.addRadioButtonGroup("Select Analysis Channel 1 ", items1, 1, 3, "2");

Dialog.addRadioButtonGroup("Select Analysis Channel 2", items1, 1, 3, "3");

Dialog.addRadioButtonGroup("Select Cell Mask Channel", items1, 1, 3, "2");

Dialog.addString("Nucleus Detection Threshold", Maxima);

Dialog.addString("Scale Bar Size (uM)", Scale);

Dialog.addRadioButtonGroup("Filetype", items2, 1, 3, ".nd2");

Dialog.addRadioButtonGroup("Image Crop/Vignette Removal", items4, 1, 3, "Yes");

Dialog.addRadioButtonGroup("Analysis Method", items3, 1, 3, "Manual Thresholding");

Dialog.addMessage("For Maximum Intensity Mode, Set Pixel Value Cut-offs:");

Dialog.addString("Channel 1 Fluorescence Max Cut-off", Sensitivity1);

Dialog.addString("Channel 2 Fluorescence Max Cut-off", Sensitivity1);

Dialog.addMessage("This Macro was written by: \nChad J. Johnson, \nMonash Micro Imaging, \nMonash University, \nMelbourne, Australia.\nCopyright: September 2020.\nIf you use this macro for your research please reference as appropriate.");

Dialog.show();

Nucleus = Dialog.getRadioButton();

Analysis1 = Dialog.getRadioButton();

Analysis2 = Dialog.getRadioButton();

CellMask = Dialog.getRadioButton();

Maxima = Dialog.getString();

Scale = Dialog.getString();

Filetype = Dialog.getRadioButton();

Vignette = Dialog.getRadioButton();

Method = Dialog.getRadioButton();

Sensitivity1 = Dialog.getString();

Sensitivity2 = Dialog.getString();

//End Dialogue

dir1 = getDirectory("Choose Image Directory");

StackFolder = File.exists(dir1+"Results");

if (StackFolder==0) {

dir2 = File.makeDirectory(dir1+"Results");

}

if (StackFolder==1) {

dir2 = dir1+"Results/";

}

list = getFileList(dir1);

setBatchMode(false);

for (i=0; i<list.length; i++) {

showProgress(i, list.length);

filename = dir1 + list[i];

/*if (BatchMode == 0){

setBatchMode(false);

}else{

waitForUser("Analysis will now be done as a background process. Please wait");

setBatchMode(true);

}*/

if (endsWith(filename, Filetype)) {

run("Bio-Formats Windowless Importer", "open=["+filename+"]");

roiManager("Reset");

setSlice(Analysis1);

run("Green");

setSlice(Analysis2);

run("Red");

OriginalImage = getTitle();

if (Vignette == "Yes"&&VignetteCounter == 0&&nImages >= 1){

getDimensions(width, height, channels, slices, frames);

makeRectangle((width/10),(height/10),(width*0.8),(height*0.8));

waitForUser("Set area to crop for vignette removal. This area will be used for all other images");

getSelectionBounds(x, y, width2, height2);

run("Crop");

run("Select None");

}

if (Vignette == "Yes"&&VignetteCounter == 1){

makeRectangle(x, y, width2, height2);

run("Crop");

run("Select None");

}

VignetteCounter = 1;

run("Duplicate...", "duplicate");

Image = getTitle();

run("Split Channels");

selectWindow("C"+Nucleus+"-"+Image);

NucleusChannel = getTitle();

selectWindow("C"+Analysis1+"-"+Image);

AnalysisChannel1 = getTitle();

run("Duplicate...", " ");

Output1 = getTitle();

selectWindow("C"+Analysis2+"-"+Image);

AnalysisChannel2 = getTitle();

run("Duplicate...", " ");

Output2 = getTitle();

selectWindow("C"+CellMask+"-"+Image);

run("Duplicate...", " ");

CellMaskChannel = getTitle();

selectWindow(NucleusChannel);

run("Enhance Local Contrast (CLAHE)", "blocksize=127 histogram=256 maximum=3 mask=*None* fast_(less_accurate) process_as_composite");

run("Find Maxima...", "prominence="+Maxima+" output=[Segmented Particles]");

SegmentedNucleus = getTitle();

close(NucleusChannel);

selectWindow(CellMaskChannel);

run("Enhance Local Contrast (CLAHE)", "blocksize=127 histogram=256 maximum=3 mask=*None* fast_(less_accurate) process_as_composite");

run("Gaussian Blur...", "sigma=4");

if (ThreshTrigger == 0){

ThreshTrigger = 1;

run("Threshold...");

setAutoThreshold("Mean dark");

waitForUser("Set Threshold of Cell Margin. Click OK to continue");

getThreshold(lower, upper);

}else{

setThreshold(lower, upper);

}

run("Measure");

TissueArea = getResult("Area");

close("Results");

selectWindow(CellMaskChannel);

run("Convert to Mask");

run("Invert");

imageCalculator("Subtract", SegmentedNucleus, CellMaskChannel);

Segmented = getTitle();

selectWindow(Segmented);

run("Analyze Particles...", "size=10-Infinity exclude add");

roiManager("save", dir1+"Results/All_ROIs.zip");

close(SegmentedNucleus);

close(Segmented);

close(CellMaskChannel);

ROICOUNT = roiManager("count");

TotalCells = roiManager("count");

if (Method == "Manual Thresholding"){

Analysis_Green_Threshold();

Analysis_Red_Threshold();

Analysis_GreenRed_Threshold();

}

if (Method == "Maximum Intensity"){

Analysis_Green_Max();

Analysis_Red_Max();

Analysis_GreenRed_Max();

}

//Prepare a log table for the data to be logged

TableTitle= "Cell Analysis";

TableTitle2="["+TableTitle+"]";

Results = isOpen(TableTitle);

if (Results==true) {

selectWindow(TableTitle);

close("Results");

}else{

run("Table...", "name="+TableTitle2+" width=600 height=250");

print(TableTitle2, "\\Headings:Image Name\tTotal Number of Cells\t Green Positive\tGreen Negative\tPercentage Green Positive\tGreen Fluorescence Cut-off\tRed Positive\tRed Negative\tPercentage Red Positive\tRed Fluorescence Cut-off\tGreen/Red Positive\tGreen/Red Negative\tPercentage Green/Red Positive");

}

print(TableTitle2, OriginalImage+ "\t" +TotalCells+ "\t" +GPositive+ "\t" +GNegative+ "\t" +GPercPos+ "\t" +Sensitivity1+ "\t" +RPositive+ "\t" +RNegative+ "\t" +RPercPos+ "\t" +Sensitivity2+ "\t" +GRPositive+ "\t" +GRNegative+ "\t" +GRPercPos);

BatchMode = 1;

}

}

close("log");

waitForUser("Analysis Complete. Please save results");

function Analysis_Green_Max(){

roiManager("reset");

roiManager("open", dir1+"Results/All_ROIs.zip");

selectWindow(Output1);

roiManager("Show None");

roiManager("Show All");

GPositive = 0;

GNegative = 0;

for (k = 0; k < ROICOUNT; k++) {

roiManager("Select", ROICOUNT-k-1);

run("Measure");

Max = getResult("Max");

if (Max >= Sensitivity1){

roiManager("Set Color", "green");

GPositive = GPositive + 1;

}else{

roiManager("delete");

GNegative = GNegative + 1;

}

}

selectWindow(Output1);

run("RGB Color");

roiManager("Show None");

roiManager("Show All without labels");

run("Flatten");

saveAs("Jpeg", dir2+OriginalImage+"_Green_Output");

close();

roiManager("reset");

GPercPos = (GPositive/TotalCells)*100;

GCellsPerArea = GPositive/TissueArea;

run("Collect Garbage");

close("Results");

}

function Analysis_Red_Max(){

roiManager("reset");

roiManager("open", dir1+"Results/All_ROIs.zip");

selectWindow(Output2);

roiManager("Show None");

roiManager("Show All");

RPositive = 0;

RNegative = 0;

for (l = 0; l < ROICOUNT; l++) {

roiManager("Select", ROICOUNT-l-1);

run("Measure");

Max = getResult("Max");

if (Max >= Sensitivity2){

roiManager("Set Color", "red");

RPositive = RPositive + 1;

}else{

roiManager("delete");

RNegative = RNegative + 1;

}

}

selectWindow(Output2);

run("RGB Color");

roiManager("Show None");

roiManager("Show All without labels");

run("Flatten");

saveAs("Jpeg", dir2+OriginalImage+"_Red_Output");

close();

roiManager("reset");

RPercPos = (RPositive/TotalCells)*100;

RCellsPerArea = RPositive/TissueArea;

run("Collect Garbage");

close("Results");

}

function Analysis_GreenRed_Max(){

roiManager("reset");

roiManager("open", dir1+"Results/All_ROIs.zip");

selectWindow(OriginalImage);

roiManager("Show None");

roiManager("Show All");

GRPositive = 0;

GRNegative = 0;

for (m = 0; m < ROICOUNT; m++) {

roiManager("Select", ROICOUNT-m-1);

setSlice(Analysis1);

run("Measure");

Max1 = getResult("Max");

setSlice(Analysis2);

run("Measure");

Max2 = getResult("Max");

if (Max1 >= Sensitivity1&&Max2 >= Sensitivity2){ //Both G and R positive

roiManager("Set Color", "yellow");

GRPositive = GRPositive + 1;

}else{

//Do Nothing

}

if (Max1 >= Sensitivity1&&Max2 < Sensitivity2){ //Only G positive

roiManager("Set Color", "green");

GRNegative = GRNegative + 1;

}else{

//Do Nothing

}

if (Max2 >= Sensitivity2&&Max1 < Sensitivity1){ //Only R positive

roiManager("Set Color", "red");

GRNegative = GRNegative + 1;

}else{

//Do Nothing

}

if (Max2 < Sensitivity2&&Max1 < Sensitivity1){ //Both G and R Negative

roiManager("Set Color", "magenta");

GRNegative = GRNegative + 1;

}else{

//Do Nothing

}

}

selectWindow(OriginalImage);

run("Make Composite");

Composite = getTitle();

run("RGB Color");

Final = getTitle();

roiManager("Show None");

roiManager("Show All without labels");

run("Flatten");

Final2 = getTitle();

run("Scale Bar...", "width="+Scale+" height=8 font=28 color=White background=None location=[Lower Right] bold overlay");

saveAs("Jpeg", dir2+OriginalImage+"_Green_Red_Output");

close(Composite);

close(Final);

close(Image);

close(OriginalImage);

close(Output1);

close(Output2);

close(AnalysisChannel1);

close(AnalysisChannel2);

close(Final2);

close("log");

close("Results");

roiManager("reset");

GRPercPos = (GRPositive/TotalCells)*100;

GRCellsPerArea = GRPositive/TissueArea;

run("Collect Garbage");

close("Results");

File.delete(dir1+"Results/All_ROIs.zip");

}

function Analysis_Green_Threshold(){

roiManager("reset");

roiManager("open", dir1+"Results/All_ROIs.zip");

selectWindow(Output1);

if (ThreshTrigger2 == 0){

ThreshTrigger2 = 1;

run("Threshold...");

setAutoThreshold("Intermodes dark");

waitForUser("Set Detection Threshold for the First Analysis Channel");

getThreshold(lower2, upper2);

}else{

setThreshold(lower2, upper2);

}

setOption("BlackBackground", true);

run("Convert to Mask");

Output1Mask = getTitle();

roiManager("Show None");

roiManager("Show All");

GPositive = 0;

GNegative = 0;

for (k = 0; k < ROICOUNT; k++) {

roiManager("Select", ROICOUNT-k-1);

run("Measure");

Max = getResult("Max");

if (Max == 255){

roiManager("Set Color", "green");

GPositive = GPositive + 1;

}else{

roiManager("delete");

GNegative = GNegative + 1;

}

}

selectWindow(Output1);

run("RGB Color");

roiManager("Show None");

roiManager("Show All without labels");

run("Flatten");

saveAs("Jpeg", dir2+OriginalImage+"_Green_Output");

close();

roiManager("reset");

GPercPos = (GPositive/TotalCells)*100;

GCellsPerArea = GPositive/TissueArea;

run("Collect Garbage");

close("Results");

}

function Analysis_Red_Threshold(){

roiManager("reset");

roiManager("open", dir1+"Results/All_ROIs.zip");

selectWindow(Output2);

if (ThreshTrigger3 == 0){

ThreshTrigger3 = 1;

run("Threshold...");

setAutoThreshold("Intermodes dark");

waitForUser("Set Detection Threshold for the Second Analysis Channel");

getThreshold(lower3, upper3);

}else{

setThreshold(lower3, upper3);

}

setOption("BlackBackground", true);

run("Convert to Mask");

Output2Mask = getTitle();

roiManager("Show None");

roiManager("Show All");

RPositive = 0;

RNegative = 0;

for (k = 0; k < ROICOUNT; k++) {

roiManager("Select", ROICOUNT-k-1);

run("Measure");

Max = getResult("Max");

if (Max == 255){

roiManager("Set Color", "red");

RPositive = RPositive + 1;

}else{

roiManager("delete");

RNegative = RNegative + 1;

}

}

selectWindow(Output2);

run("RGB Color");

roiManager("Show None");

roiManager("Show All without labels");

run("Flatten");

saveAs("Jpeg", dir2+OriginalImage+"_Red_Output");

close();

roiManager("reset");

RPercPos = (RPositive/TotalCells)*100;

RCellsPerArea = RPositive/TissueArea;

run("Collect Garbage");

close("Results");

}

function Analysis_GreenRed_Threshold(){

roiManager("reset");

roiManager("open", dir1+"Results/All_ROIs.zip");

selectWindow(OriginalImage);

roiManager("Show None");

roiManager("Show All");

GRPositive = 0;

GRNegative = 0;

selectWindow(Output2Mask);

rename("OutputMask2");

Output2Mask = getTitle();

selectWindow(Output1Mask);

rename("OutputMask1");

Output1Mask = getTitle();

run("Images to Stack", "name=Stack title=OutputMask");

AnalysisStack = getTitle();

for (m = 0; m < ROICOUNT; m++) {

selectWindow(AnalysisStack);

setSlice(1);

roiManager("Select", ROICOUNT-m-1);

run("Measure");

Max1 = getResult("Max");

setSlice(2);

roiManager("Select", ROICOUNT-m-1);

run("Measure");

Max2 = getResult("Max");

if (Max1 == 255&&Max2 == 255){ //Both G and R posituve

roiManager("Set Color", "yellow");

GRPositive = GRPositive + 1;

}else{

//Do Nothing

}

if (Max1 == 255&&Max2 == 0){ //Only G positive

roiManager("Set Color", "green");

GRNegative = GRNegative + 1;

}else{

//Do Nothing

}

if (Max2 == 255&&Max1 == 0){ //Only R positive

roiManager("Set Color", "red");

GRNegative = GRNegative + 1;

}else{

//Do Nothing

}

if (Max1 == 0&&Max2 == 0){ //Both G and R Negative

roiManager("Set Color", "magenta");

GRNegative = GRNegative + 1;

}else{

//Do Nothing

}

}

selectWindow(OriginalImage);

run("Make Composite");

Composite = getTitle();

run("RGB Color");

Final = getTitle();

roiManager("Show None");

roiManager("Show All without labels");

run("Flatten");

Final2 = getTitle();

run("Scale Bar...", "width="+Scale+" height=8 font=28 color=White background=None location=[Lower Right] bold overlay");

saveAs("Jpeg", dir2+OriginalImage+"_Green_Red_Output");

close(Composite);

close(Final);

close(Image);

close(OriginalImage);

close(Output1);

close(Output2);

close(AnalysisChannel1);

close(AnalysisChannel2);

close(Final2);

close(AnalysisStack);

close("log");

close("Results");

roiManager("reset");

GRPercPos = (GRPositive/TotalCells)*100;

GRCellsPerArea = GRPositive/TissueArea;

run("Collect Garbage");

close("Results");

File.delete(dir1+"Results/All_ROIs.zip");

}
